# Egg freezing for fertility preservation and family planning: a nationwide survey of US Obstetrics and Gynecology residents

**DOI:** 10.1186/s12958-019-0459-x

**Published:** 2019-01-29

**Authors:** Navid Esfandiari, Julia Litzky, Joshua Sayler, Pavel Zagadailov, Karen George, Leslie DeMars

**Affiliations:** 1 Department of Obstetrics and Gynecology, Dartmouth Hitchcock Medical Center, One Medical Center Drive, Lebanon, NH 03765 USA; 2 Geisel School of Medicine at Dartmouth, 1 Rope Ferry Road, Hanover, NH 03756 USA; 3 Department of Obstetrics and Gynecology, The George Washington Medical Faculty Associates, 4920 Elm Street, Bethesda, MD 20814 USA

**Keywords:** Elective egg freezing, Residency, Fertility preservation, REI rotation, Family planning

## Abstract

**Background:**

Little is known about resident attitudes toward elective egg freezing (EF) or how educational exposure to EF affects residents’ views and ability to counsel patients. This study aimed to evaluate US OB/GYN residents’ views on elective EF, decisions regarding family planning, and whether education on EF affects these views and self-reported comfort discussing EF with patients.

**Methods:**

A 32 question survey was emailed to program directors at all US residency programs for distribution to residents. Chi-square tests were used to evaluate the relationship between educational factors and views on EF and comfort counselling patients.

**Results:**

Of those surveyed, 106 residents and 7 fellows completed the survey (103 female). Almost three quarters of female respondents reported postponing pregnancy due to residency (71.8%). Non-exclusive reasons for this choice included career plans (54.4%) and concern for childcare (51.5%) and for fellow residents and their program (50.5%). Of the male and female residents who reported educational exposure to EF (57.5%), almost all of them (95.4%) received this in an REI rotation. Only half of female residents reported being comfortable counseling a patient on EF (49.5%). For female residents, education on EF (*p* = 0.03) and more advanced level of residency (*p* = 0.02) were significantly associated with comfort counseling a patient on EF.

**Conclusions:**

Female OB/GYN residents are choosing to delay pregnancy during residency for career and social support reasons. Few residents feel comfortable counseling patients on EF, but appropriate curricular content on EF during residency could improve residents’ comfort in assisting patients with reproductive planning.

**Electronic supplementary material:**

The online version of this article (10.1186/s12958-019-0459-x) contains supplementary material, which is available to authorized users.

## Background

Oocyte cryopreservation technologically has advanced significantly in the last three decades, and currently is considered an adjunct technique in fertility treatment all around the world. Oocyte cryopreservation initially was developed as a fertility sparing option for women undergoing cancer therapy or requiring bilateral oophorectomy for a medical condition. Most oncologic societies now recommend pre-treatment discussion of fertility preservation for women with cancer who are of childbearing age. More recently, the increasing age of first elective pregnancy and age-related infertility/subfertility has led to interest in and use of elective oocyte cryopreservation, referred to here as egg freezing (EF), among younger, healthy women. In Oct. 2012, egg freezing was reclassified from an experimental procedure to an elective procedure by the American Society for Reproductive Medicine [1], making EF available as a means of fertility preservation for women hoping to delay childbearing.

Currently, little is known about societal attitudes toward EF, in particular what makes women in favor of or against the procedure both ethically and personally. Surveys of the general population indicate that women who were 30–45 years old, had personally experienced infertility, and had higher levels of education had more favorable views of elective EF procedures [2]. Understanding how patients acquire knowledge regarding EF and what determines whether physicians have these conversations with interested patients is essential in improving informed decision on egg freezing.

Despite their central role in counseling patients on EF, little is known about medical trainees’ views on the procedure. Among medical students and house staff with prior knowledge of EF, 80% reported acquiring this knowledge through their formal education [3], indicating that EF is not well-covered in medical schools. A survey of U.S. Obstetrics and Gynecology (OB/GYN) residents indicated that 83% felt that they should begin conversations with their patients regarding fertility, and most felt that these conversations should occur during annual exams [4]. This survey also indicated that residents were under-informed about fertility; 47% overestimated the age of marked decline in female fertility, and over three quarters overestimated IVF success rates [4].

Additionally, medical professionals, especially residents, could be a target population for EF, as they are young individuals who may be delaying pregnancy until after they complete their residencies [5]. Given their medical background, residents are likely to be knowledgeable about EF or can easily learn about it, allowing them to make well-informed decisions regarding the procedure. However, although residents and medical students report delaying pregnancy [5], the reasons for doing so, and particularly how this may affect their views on EF, are not well-established. To evaluate resident views on EF and how education on EF impacts their views and practice, we surveyed US OB/GYN residents to determine 1) whether they intentionally delay having children during residency, 2) their reasons for this delay, 3) the extent of their knowledge about oocyte freezing and 4) how educational exposure to EF affects their views on oocyte freezing.

## Methods

### Survey design and distribution

A questionnaire was prepared by a broad team of researchers at a US academic medical center representing Reproductive Endocrinology and Infertility, Gynecologic Oncology and General Obstetrics and Gynecology. The number of questions, selection of answers, and appropriateness of the questions were reviewed during a presentation to OB/GYN faculty members. Feedback was used to modify the content and order of the questions before the survey was sent out. The study was approved by the institution’s Committee for Protection of Human Subjects (CPHS). The online survey was created using Wufoo, a secure online form builder designed by SurveyMonkey [6]. In March 2017, the survey was sent via email to all OB/GYN program directors across the United States and they were asked to distribute it to their OB/GYN residents and fellows from all training years.

The survey was anonymous and the CPHS determined that no consent was necessary. Participants were not provided any incentive for completing the survey. The final questionnaire consisted of 32 items with 4 subcategories including demographics (e.g. age, ethnicity, sexual orientation), description of their residency program (e.g. university or community based, onsite IVF program, self-reported educational exposure to EF), residents’ views on egg freezing (e.g. would they consider it for themselves, what factors would make them more likely to consider it), and residents’ family planning decisions (e.g. have they delayed childbearing due to residency, if and when they plan to have their first child or additional children) [see Additional file 1]. There were 4 questions (numbers 21–24) that only female respondents could see and answer. Questions were a combination of multiple choice, yes/no, and short answer questions. Responses were collected through the end of March 2017.

### Cohort selection

In total, 114 residents responded to the survey. This was a lower than expected response rate. Of these, 1 was excluded for not reporting gender or year in residency. This left 103 female (91.2%) and 10 male residents and fellows. This cohort was used to generate demographic and descriptive statistics. For statistical analysis, the cohort of female residents was used (*n* = 103). Four participants were removed from this cohort due to missing data on the variables of interest (whether they think residents should consider EF, whether they would personally consider EF, and/or the length of the Reproductive Endocrinology and Infertility (REI) rotation at their institution), which left 99 female participants.

### Statistical analysis

All statistics were performed in R 3.4.2. Descriptive statistics were used to examine demographics, educational background and exposures, and residents’ views on EF. Chi square tests were used to evaluate the relationships between educational exposure to elective EF and female residents’ comfort in counseling patients and personal interest in elective EF. The educational exposure variables explored were year in residency, self-reported formal education in EF, whether their residency program had an IVF program, the length of the REI rotation at their institution, and the length of their REI rotation for those residents who had done an REI rotation. Outcome variables included whether residents felt comfortable counseling patients on elective EF, whether they would personally consider EF, and whether they thought all female residents should consider EF.

## Results

### Cohort demographics

Overall, the majority of participants (66%) were aged 26–30, with only 6 respondents age 35 or over (Table 1). Most were Caucasian (73.8%), married (58.2%), and heterosexual (92.2%). Respondents were mostly in residency programs in the Great Lakes region (31.1%) and Northeast (24.3%), but responses were received from all over the country, though only one response was received from the Northwest and 9 from the Southwest. The majority of participants (91.3%) had an MD degree and the other 8.7% had a DO, and 14.6% had an additional PhD, MSc, or MPH degree. Residents were mostly evenly distributed across year of residency, with slightly more participants in year 2 (*n* = 28, 27.2%) and fewer in year 4 (*n* = 19, 18.4%). Seven fellows responded, 4 of which were maternal-fetal medicine fellows.

**Table 1:** Demographic information for male and female residents/fellows, including educational background. All numbers are presented as N (%)

	Female	Male	All
	*n* = 103	*n* = 10	*n* = 113
Age			
*26–30*	68 (66.0)	6 (60.0)	74 (65.5)
*31–34*	29 (28.2)	4 (40.0)	33 (29.2)
*> = 35*	6 (5.8)	0 (0.0)	6 (5.3)
Ethnicity			
*African-American*	7 (6.8)	0 (0.0)	7 (6.2)
*Asian/Pacific Islander*	7 (6.8)	0 (0.0)	7 (6.2)
*American Indian/Alaska Native*	0 (0.0)	1 (10.0)	1 (0.9)
*Caucasian*	76 (73.8)	8 (80.0)	84 (74.3)
*Hispanic/Latino*	9 (8.7)	1 (10.0)	10 (8.8)
*Multiracial*	2 (1.9)	0 (0.0)	2 (1.8)
*Rather Not Say*	2 (1.9)	0 (0.0)	2 (1.8)
Relationship Status			
*Single*	24 (23.3)	3 (30.0)	27 (23.9)
*Married*	60 (58.2)	6 (60.0)	66 (58.4)
*Living with Partner*	19 (18.4)	1 (10.0)	20 (17.7)
*Divorced or widowed*	0 (0.0)	0 (0.0)	0 (0.0)
Sexuality			
*Heterosexual*	95 (92.2)	9 (90.0)	104 (92.0)
*Homosexual*	4 (3.9)	1 (10.0)	5 (4.4)
*Bisexual*	4 (3.9)	0 (0.0)	4 (3.5)
US Region			
*Great Lakes Region*	32 (31.1)	2 (20.0)	34 (30.1)
*Northeast Region*	25 (24.3)	2 (20.0)	27 (23.9)
*Mid-Atlantic Region*	16 (15.5)	2 (20.0)	18 (15.9)
*Southwest Region*	10 (9.7)	0 (0.0)	10 (8.8)
*Gulf Coast Region*	9 (8.7)	1 (10.0)	10 (8.8)
*Southeast Region*	8 (7.8)	1 (10.0)	9 (8.0)
*Northwest Region*	1 (1.0)	0 (0.0)	1 (0.9)
*Missing*	2 (1.9)	2 (20.0)	4 (3.5)
Educational Background			
Degree (non-exclusive)			
*MD*	94 (91.3)	10 (100.0)	104 (92.0)
*DO*	9 (8.7)	0 (0.0)	9 (8.0)
*PhD*	1 (1.0)	1 (10.0)	2 (1.8)
*MSc/MPH*	14 (13.6)	3 (30.0)	17 (15.0)
Year of Training			
*PGY-1*	22 (21.4)	3 (30.0)	25 (22.1)
*PGY-2*	28 (27.2)	3 (30.0)	31 (27.4)
*PGY-3*	27 (26.2)	3 (30.0)	30 (26.5)
*PGY-4*	19 (18.4)	1 (10.0)	20 (17.7)
*Fellow*	7 (6.8)	0 (0.0)	7 (6.2)
Fellowship Type			
*Maternal Fetal Medicine*	4 (3.9)	0 (0.0)	4 (3.5)
*Pelvic Medicine and Reconstructive Surgery*	1 (1.0)	0 (0.0)	1 (0.9)
*Gyn/Onc*	1 (1.0)	0 (0.0)	1 (0.9)
*REI*	1 (1.0)	0 (0.0)	1 (0.9)

### Residency programs and educational exposure to EF

Most residents were in a university-based program (84.1%) and reported their program as having an IVF program on-site (Table 2). Of those with an IVF program (*n* = 95), 63.1% (*n* = 57) reported availability of oocyte freezing on-site. However, almost a third of residents (30.5%, *n* = 29) who reported that their department has an IVF program were unsure regarding oocyte freezing availability. Similarly, many female residents were unsure if their employers offered oocyte freezing as a benefit (39.8%), and only 17.5% reported that there was such a benefit offered.

**Table 2:** Residency program descriptors and status of education on elective oocyte freezing. All numbers are presented as N (%)

	Female	Male	All
	*n* = 103	*n* = 10	*n* = 113
Type of Program			
*University hospital based*	86 (83.5)	9 (90.0)	95 (84.1)
*Community hospital based*	15 (14.6)	1 (10.0)	16 (14.2)
*Other*	2 (1.9)	0 (0.0)	2 (1.8)
Does your department have an IVF program?			
*Yes*	87 (84.5)	8 (80.0)	95 (84.1)
*No*	11 (10.7)	2 (20.0)	13 (11.5)
*Unsure*	5 (4.9)	0 (0.0)	5 (4.4)
If yes, does your department offer on-site oocyte freezing?			
*Yes*	54 (52.4)	6 (60.0)	60 (53.1)
*No*	5 (4.9)	1 (10.0)	6 (5.3)
*Unsure*	28 (27.2)	1 (10.0)	29 (25.7)
*No IVF program or unsure if IVF program*	16 (15.5)	2 (20.0)	18 (15.9)
Does your employer offer oocyte-freezing as a benefit?			
*Yes*	18 (17.5)	–	–
*No*	43 (41.7)	–	–
*Unsure*	41 (39.8)	–	–
*Missing*	1 (1.0)	–	–
Do you receive education on oocyte freezing?			
*Yes*	61 (59.2)	4 (40.0)	65 (57.5)
*No*	42 (40.8)	6 (60.0)	48 (42.5)
If so, where did you receive that education? (non-exclusive)			
*REI rotation*	58 (56.3)	4 (40.0)	62 (54.9)
*Didactics*	19 (18.4)	2 (20.0)	21 (18.6)
*Grand rounds*	11 (10.7)	1 (10.0)	12 (10.6)
*Oncology rotation*	4 (3.9)	1 (10.0)	5 (4.4)
*Benign gynecology rotation*	2 (1.9)	1 (10.0)	3 (2.7)
*Other*	2 (1.9)	0 (0.0)	2 (1.8)
How long is the REI rotation at your program?			
*No REI rotation*	3 (2.9)	1 (10.0)	4 (3.5)
*1 to 4 weeks*	17 (16.5)	2 (20.0)	19 (16.8)
*5 to 8 weeks*	45 (43.7)	3 (30.0)	48 (42.5)
*9 to 12 weeks*	27 (26.2)	3 (30.0)	30 (26.5)
*13 to 16 weeks*	5 (4.9)	0 (0.0)	5 (4.4)
*17 to 20 weeks*	3 (2.9)	1 (10.0)	4 (3.5)
*Missing*	3 (2.9)	0 (0.0)	3 (2.7)

Cells containing “—” are responses to questions that male participants were not asked to complete, and therefore responses are only presented for female participants

Only a little over half of respondents reported receiving formal education on EF (57.5%). Of those who did (*n* = 65), almost all (95.4%) received this in an REI rotation. Responses were non-exclusive, and residents who received EF education also reported EF exposure during didactics (33.8%), grand rounds (18.4%), and benign gynecology (4.6%) and oncology (7.7%) rotations. Most residents reported having an REI rotation at their residency program (93.8%). Of those with an REI program (*n* = 106), most were 5–8 weeks (45.2%) or 9 to 12 weeks (28.3%). However, 17.9% of residents with an REI rotation reported that their REI rotation was only 1 to 4 weeks long.

### Resident views on counseling patients regarding EF

About half of female residents reported being comfortable counseling a patient on oocyte freezing (49.5%), with 19.4% being unsure (Table 3). When asked what they felt was the most important information for a prospective patient, female residents were most concerned with costs, with 98.1% indicating that they felt cost of a cycle was important, and 90.3% identifying storage fees as one of the most important factors (answers were non-exclusive). Female residents also frequently indicated concern for knowing the optimal age for EF (90.3%), the length of time which oocytes are viable (89.3%), and how many oocytes are needed to obtain one pregnancy (79.6%).

**Table 3:** Resident views of elective oocyte freezing. All numbers are presented as N (%)

	Female	Male	All
	*n* = 103	*n* = 10	*n* = 113
Views on elective oocyte freezing for patients			
Do you feel comfortable counseling a patient on oocyte freezing?			
*Yes*	51 (49.5)	–	–
*No*	32 (31.1)	–	–
*Unsure*	20 (19.4)	–	–
What information do you consider most important to know as a prospective patient? (nonexclusive)			
*How much does an oocyte-freezing cycle cost?*	101 (98.1)	5 (50.0)	106 (93.8)
*What is the optimal age for oocyte freezing?*	93 (90.3)	9 (90.0)	102 (90.3)
*What are the annual storage fees for vitrified oocytes?*	93 (90.3)	5 (50.0)	98 (86.7)
*How many years are vitrified oocytes viable?*	92 (89.3)	5 (50.0)	97 (85.8)
*What is the average number of oocytes needed to obtain one pregnancy?*	82 (79.6)	7 (70.0)	89 (78.8)
*Are there health reasons to consider oocyte-freezing at a younger age?*	61 (59.2)	6 (60.0)	67 (59.3)
*What happens if my vitrified oocytes are lost due to error?*	36 (35.0)	8 (80.0)	44 (38.9)
*Can I donate unused vitrified oocytes to research?*	30 (29.2)	1 (10.0)	31 (27.4)
*Can I donate unused vitrified oocytes to another woman?*	26 (25.2)	1 (10.0)	27 (23.9)
*Why do women freeze their oocytes?*	24 (23.3)	4 (40.0)	28 (24.8)
Views on elective oocyte freezing for residents and themselves			
Should all female residents consider oocyte freezing?			
*Yes*	27 (26.2)	–	–
*No*	57 (55.3)	–	–
*Unsure*	18 (17.5)	–	–
*Missing*	1 (1.0)	–	–
Would you consider freezing your oocytes?			
*Yes*	47 (45.6)	–	–
*No*	39 (37.9)	–	–
*Unsure*	16 (15.5)	–	–
*Missing*	1 (1.0)	–	–
If so, at what age would you do so?			
*25 or under*	3 (2.9)	–	–
*26–30*	33 (32.0)	–	–
*31–34*	42 (40.8)	–	–
*35–37*	12 (11.7)	–	–
*38–40*	4 (3.9)	–	–
*Unsure*	3 (2.9)	–	–
*Missing*	6 (5.8)	–	–
Would you consider freezing your oocytes if you did not have a partner?			
*Yes*	63 (61.2)	–	–
*No*	20 (19.4)	–	–
*Unsure*	16 (15.5)	–	–
*Missing*	4 (3.9)	–	–
What do you consider an affordable out-of-pocket cost for oocyte freezing?			
*$2000–$5000*	70 (68.0)	–	–
*$5000–$10,000*	19 (18.4)	–	–
*$10,000–$15,000*	1 (1.0)	–	–
*Unsure*	13 (12.6)	–	–
Would you prefer to be employed by an employer who offers oocyte freezing as part of benefits package?			
*Yes*	67 (65.0)	–	–
*No*	13 (12.6)	–	–
*Unsure*	23 (2.3)	–	–
Would you consider freezing your oocytes in the following circumstances? (nonexclusive)			
*There is insurance coverage or employer financial support for the cycle*	76 (73.8)	7 (70.0)	83 (73.5)
*There is no effect on health of children born from vitrified oocytes*	61 (59.2)	5 (50.0)	66 (58.4)
*You did not currently have a partner*	62 (60.2)	3 (30.0)	65 (57.5)
*You currently don’t have children*	55 (53.4)	5 (50.0)	60 (53.1)
*There is higher pregnancy rate using vitrified oocytes than with natural or routine IVF treatment at advanced female age*	52 (50.5)	4 (40.0)	56 (49.6)
*Oocyte-freezing was available locally*	41 (39.8)	4 (40.0)	45 (39.8)
If you have a partner would you be more interested in freezing embryos than eggs?			
*Yes*	49 (47.6)	5 (50.0)	54 (47.8)
*No*	41 (39.8)	3 (30.0)	44 (38.9)
*Missing*	13 (12.6)	2 (20.0)	15 (13.3)

Cells containing “—” are responses to questions that male participants were not asked to complete, and therefore responses are only presented for female participants

### Resident choices and views regarding family planning

The majority of surveyed male and female residents had no children at the time of the survey (83.2%), and most planned to have children in the future (85.8%) (Table 4). Of those planning to have children in the future, most planned to have their next/first child at ages 31–34 (63.7%). Importantly, 71.8% of female respondents reported intention to delay pregnancy during residency. Participants were provided with a list of residency-related reasons for delaying pregnancy and selected all that applied to their decision-making process. Among women, career plans were the most frequently cited reason (54.4%). Over half of those who reported delaying pregnancy cited concern over availability of childcare as a significant reason (51.5%), and a similar proportion cited concern for their fellow residents and residency program due to maternity leave (50.5%). Residents were also noted concern for the lack of benefits offered by their program or institution (30.1%) and for prenatal health (16.5%). Female residents were also given the option to self-report reasons for delaying pregnancy, which could be categorized into three main groups: lack of time (12.6%), lack of a partner (5.8%), and concern for finances (4.9%).

**Table 4:** Resident family planning decisions and reasons for delaying pregnancy. All numbers are presented as N (%)

	Female	Male	All
	*n* = 103	*n* = 10	*n* = 113
Number of Children			
*None*	85 (82.5)	9 (90.0)	94 (83.2)
*One*	14 (13.6)	0 (0.0)	14 (12.4)
*Two*	3 (2.9)	0 (0.0)	3 (2.7)
*Three*	1 (1.0)	1 (10.0)	2 (1.8)
Do you plan to have a/another child			
*Yes*	88 (85.4)	9 (90.0)	97 (85.8)
*No*	4 (3.9)	0 (0.0)	4 (3.5)
*Undecided*	8 (.8)	1 (10.0)	9 (8.0)
*Missing*	3 (2.9)	0 (0.0)	3 (2.7)
At what age to you plan to have a/another child?			
*26–30*	16 (15.5)	1 (10.0)	17 (15.0)
*31–34*	68 (66.0)	4 (40.0)	72 (63.7)
*35–37*	12 (11.7)	5 (50.0)	17 (15.0)
*38–40*	1 (1.0)	0 (0.0)	1 (0.9)
*41–42*	2 (1.9)	0 (0.0)	2 (1.8)
*Not planning on having a/another child*	4 (3.9)	0 (0.0)	4 (3.5)
Have you postponed pregnancy due to your residency?			
*Yes*	74 (71.8)	4 (44.4)	78 (69.0)
*No*	23 (22.3)	4 (44.4)	27 (23.9)
*Unsure*	6 (5.8)	1 (11.1)	7 (6.2)
If so, what about residency let to that decision? (nonexclusive)			
*Career plans*	56 (54.4)	6 (60.0)	62 (54.9)
*Concern over availability of childcare*	53 (51.5)	2 (20.0)	55 (48.7)
*Concern for fellow residents/program*	52 (50.5)	1 (10.0)	53 (46.9)
*Lack of benefits*	31 (30.1)	1 (10.0)	32 (28.3)
*Concern over prenatal health*	17 (16.5)	2 (20.0)	19 (16.8)
Other self-reported reasons, including:			
*Lack of time*	13 (12.6)	0 (0.0)	13 (11.5)
*Lack of partner*	6 (5.8)	0 (0.0)	6 (5.3)
*Concern for finances*	5 (4.9)	1 (10.0)	6 (5.3)

### Resident views on EF for themselves

When asked about their own feelings about oocyte freezing, over half of female residents indicated that they did not think all female residents should consider EF (55.3%) or were unsure (17.5%), and 53.4% indicated that they would not consider it for themselves or were unsure (Table 3). Of those who would consider EF (*n* = 47), 44.7% indicated they would do so between ages 26 and 30 years old and 34.0% indicated that they would do so between 31 and 34 years old. Having a partner affected interest in EF, as 61.2% of female residents indicated that they would consider EF if they did not have a partner. Despite the fact that the cost of EF can be well above $10,000, only one female resident reported that a cost of $10,000–$15,000 was an affordable out of pocket cost, and only 19 (18.4%) reported that $5000–$10,000 was affordable.

Male and female residents were also asked what circumstances might encourage them to consider EF. The most frequently selected circumstance was employer financial support for EF, with almost three quarters of residents selecting this response (73.5%). When asked separately, 65.0% of female residents indicated a preference to be employed by a program that offered EF as a benefit. Not currently having a partner (57.5%), knowing that there would be no effect on the children born from vitrified oocytes (58.4%) and not currently having children (53.1%) were also frequently cited circumstances that both male and female residents indicated would encourage them to consider EF for themselves or their partner. Just under half (47.8%) indicated that, if they had a partner, they would prefer freezing embryos over oocytes.

Finally, residents were given the option to provide comments or additional information regarding their views on EF. One participant highlighted the financial burden of the procedure: “I would very much like to do oocyte cryopreservation, and I’ve tried to do so, but our insurance does not cover this as an option. Additionally, my hospital was unwilling to waive the out-of-pocket fees associated with the procedure, and my cost would have been $5,700, which I cannot afford on a resident salary.” Another resident reported having had a consult but was not able to pursue EF due to a cost of $7000–$8000, but also reported being advised to wait until “a ‘better time’ because it was ‘stressful’” and felt unsupported in her desire for EF. Other residents indicated concern regarding the time needed for the procedure: “I would be concerned about the actual process of oocyte freezing. How many visits would I need to make? What are the risks? What if I got sick from OHSS and couldn’t go to work? I would need some information on those risks and I would want to know that my program would support me if I had an adverse outcome.” Finally, one resident indicated that her religious affiliation would prohibit her from receiving IVF treatment.

### Relationship between educational exposure and views on EF

To ascertain whether educational exposure affected residents’ views on EF, we evaluated whether the following variables were associated with differences in belief that female residents should consider EF, whether female participants would consider EF for themselves, and whether residents were comfortable counseling patients about EF: year in residency, prior education on EF, the length of the REI rotation at the institution, the length of the program for those who had completed it, and whether the institution had an IVF program in house. Of these, having had education on EF (*p* = 0.03) and year in residency (*p* = 0.02) were significantly associated with being more comfortable with counseling a patient on EF but there were no other associations between exposure to and education about EF and personal feelings about EF or comfort counseling patients. Given that those later in their residency are more likely to have had educational exposure to EF, we used a chi- square test to verify that there was a relationship between post-grad year and exposure to EF education (*p* = 0.002), with those later in training being more likely to have had education in EF.

## Discussion

Despite the change of egg freezing from an experimental to elective procedure in 2012, there were only 8825 elective EF cycles in United States in 2016 [7]. Although this represents an increase of about 175% since 2013, it indicates that EF is still highly underutilized for fertility preservation. As public knowledge regarding EF increases and the technique becomes more widely available, ensuring that patients have accurate information about EF is essential so that women can make informed choices. OB/GYN residents, as future attending physicians, should be comfortable and have the knowledge base and skills to accurately and impartially counsel patients on EF. Specific curricular content on EF during residency training may translate into better patient information and awareness of the option of fertility preservation and increase access to egg freezing.

In this study, we have presented some of the first data on the relationship between resident education and their views on EF. Only half of the female residents in our study reported being comfortable counseling patients on EF. Residents who report receiving educational exposure to EF were statistically significantly more likely to report being comfortable counseling a patient on EF, indicating the importance of EF curricular exposure in successfully providing this service to female patients as part of the spectrum of discussion about fertility planning at the annual visit. While REI is considered part of the core residency experience, the exposure to formal REI rotations and Assisted Reproductive Technology varies widely among residency programs. Most residency programs have 4–8 weeks of a dedicated REI rotation, while others will incorporate this experience into a bundled benign gynecology outpatient training curriculum.

Our findings might inform residency program directors about factors important to their trainees regarding personal and professional attitudes toward EF and informs curriculum development. The length of exposure to a formal REI program did not affect comfort in counseling patients in EF, indicating that brief focused REI rotations exposing residents to Assisted Reproductive Technology could help improve patient access to OB/GYN physicians who are comfortable in discussing EF. This finding supports Anspach-Will et al.’s interventional study, which found that an hour-long educational session on EF was sufficient to significantly improve medical professionals’ knowledge-based scores about EF [3] and suggests that a brief but focused curriculum in EF could help improve resident confidence in discussing EF with patients. Female residents’ input regarding what they consider important for prospective patients to know could also guide training on EF.

Although we found that educational exposure did not affect female residents’ personal views about use of EF for themselves or other residents, our study provides valuable insight into residents’ own family planning choices and, particularly, reasons for delaying childbearing. Importantly, we found that almost three quarters of female residents reported delaying pregnancy due to their residency responsibilities. Although career plans were the most common reason cited for this delay, several other factors that could be accommodated by residency programs or institutions were also highly important to female residents, including accessibility and availability of childcare and concern for the effect of a maternity leave on their program. Almost a third were concerned about insufficient maternity benefits provided by their programs. Therefore, improving maternity leave benefits, providing mechanisms to reduce the strain maternity leave might create on a residency program, and improving childcare options could be significant institutional changes that could improve residents’ quality of life and prevent the need to delay childbearing. Programs could also consider providing EF as a benefit for female residents, as 65% indicated that they would prefer to work for a program that offered EF as a benefit. These data are similar to those found in a survey of 99 female US medical students, of which almost three quarters indicated that they would consider EF if the cost was covered by their employer [8]. While this survey was limited to Obstetrics and Gynecology residents, over half of medical school graduates are now female, indicating a growing cohort of women who potentially are delaying pregnancy because of residency demands and training requirements.

Given the frequency with which female residents delay childbearing, they possibly present an ideal population for consideration of EF. Despite this, only 46% of female OB/GYN residents indicated that they would consider EF for themselves and only 26% thought that all female residents should consider EF. Rates of personal interest in EF were much lower in our cohort than those found among female US medical students [8], which may be due to the fact that our cohort contained older participants with a greater practical knowledge of EF procedures and success rates. Residents in Obstetrics and Gynecology also might be more likely to have a partner and children than those women still in medical school. Knowledge about EF might also decrease likelihood of personal interest in EF; Tan et al. found that, after providing Singaporean medical students with an educational pamphlet about EF, those who would consider EF for themselves decreased from 70 to 49% [9], which more closely matches the rates we found among female OB/GYN residents, who would be presumed to have some knowledge of EF.

Our study also provides data on what might increase the likelihood for female residents to consider EF. The most commonly cited factors include having employer financial support, knowing that there is no increased harm or risk of abnormality of a baby conceived through EF, and not currently having a partner. The factors identified in our study were similar to those identified in a survey of 500 nulliparous but presumed-fertile women in Canada, who most commonly cited cost, risk to their own health, and risks to the child born from vitrified oocytes as concerns [10]. The frequency of concerns of cost in all of these studies indicates the importance of financial support, given that an EF cycle can cost over $10,000. The majority of female residents in this study estimated $2000–$5000 as an affordable cost. Resident concerns about the health of their offspring also indicate the importance of education and additional research on the effects of EF on the infants born from those oocytes.

Although our study provides important initial findings on the significance of REI education on comfort counseling patients on EF, as well as resident decisions regarding family planning, these data represent a very small sample of US OB/GYN residents (2.2%) [11]. The on-line survey was distributed to program directors via email. Dispersion of resident surveys is variable, and we are unable to verify that it was received by the intended respondents. There is likely respondent bias at both the program director and resident level. Additionally, although we sought responses from all U.S. residency programs, programs from the Southwest and Northwest and residents of non-Caucasian race/ethnicity are under-represented in this sample, reducing the generalizability of our findings. However, the percent of minority participants in our survey (19.5% non-Caucasian, non-Asian) was representative of those in US residency programs in 2014 (18.4%) [11]. We did not receive responses from a large number of male participants, and the percentage of female participants in our study (91.2%) was higher than that in US residency programs in 2016 (82.3%) [11]. We cannot therefore make any conclusions about the views of male OB/GYN residents on EF as a population or compare the views of male and female residents. However, our cohort is similar in size and distribution to that of the only other study we have found on US OB/GYN residents, which received responses from 5% of US residents [4], similarly predominantly from the Northeast and South, and our study therefore provides a valuable addition to the sparse literature on resident views on EF.

Because of the small response rate to our survey we are unable to identify significant relationships between educational exposure and resident views and comfort discussing EF. Although we identified two significant relationships, there may be other valuable associations that we were not powered to detect. Additionally, we are not able to determine linear relationships between level of comfort in counseling patients or level of interest in EF and educational exposures as these variables were recorded as binary. Finally, future studies are needed to determine the appropriate curricular content and timing of EF in medical education to best assure that patients are given the information necessary for informed decisions about this fertility sparing option.

## Conclusions

Overall, our study indicates that residents are delaying childbearing for their careers but many are not interested in considering elective egg freezing as a means to preserve their own fertility. Additionally, residents are often uncomfortable counseling patients on elective egg freezing. However, increased educational exposure to EF could improve resident comfort in counseling patients, though it does not seem to have an impact on residents’ own views. Given the increasing use of EF and importance of resident knowledge and education on EF, our study provides important initial findings regarding the need for resident exposure to and education on EF during their residencies. Furthermore, our results provide insight into why nearly three quarters of US residents who responded to our survey choose to delay childbearing and can provide institutions with potential changes they could implement to improve resident quality of life and help to prevent the need for residents to choose between their careers and having children. Future work is needed on how to best educate residents on EF and how long such training needs to be. Additionally, our study provides direction for future research on how to improve access to and interest in EF for residents and other women planning on delaying pregnancy and how to provide better support so that residents can have children during residency and would not feel the need to delay pregnancy.

## Additional file

Additional file 1: Survey Questions. This file contains the survey used to acquire the data presented in this manuscript. (DOCX 20 kb)

## Abbreviations

CPHS: Committee for Protection of Human Subjects
EF: Egg freezing
OB/GYN: Obstetrics and gynecology
REI: Reproductive endocrinology and infertility
