# Supplementary material for: Egg freezing for fertility preservation and family planning: a nationwide survey of US Obstetrics and Gynecology residents
Source: Reprod Biol Endocrinol. 2019 Jan 29;17:16. doi: 10.1186/s12958-019-0459-x (PMC6352445; doi:10.1186/s12958-019-0459-x)
Supplement: Supplementary file 1 — Survey Questions. This file contains the survey used to acquire the data presented in this manuscript. (DOCX 20 kb) [file 12958_2019_459_MOESM1_ESM.docx]

**Additional File 1.** Survey questions. All participants answered questions 1-8 and 17-32. Following selection of gender, questions 9-16 became available for female participants.

1. How many children do you have?
   - I do not have children
   - 1
   - 2
   - 3
   - 4
   - 5 or more
2. Do you plan to have children in the future?
   - Yes
   - No
   - Not decided yet
3. At what age do you plan to have your first/next child?
   - 25 or under
   - 26-30
   - 31-34
   - 35-37
   - 38-40
   - 41-42
   - 43 or above
4. Have you felt the need to postpone pregnancy because of your residency?
   - Yes
   - No
   - Unsure

1. If so, what are your reasons for postponing pregnancy? (check all that apply)
   - Limited maternity/paternity benefits
   - Career plans
   - Concern over prenatal health
   - Concern for child care
   - Concern for fellow residents/program
   - Other reasons (please specify)
2. Have you received education in your program for oocyte freezing to preserve fertility and postpone pregnancy?
   - Yes
   - No
3. If yes, your training has been through (check all that apply):
   - Didactics
   - REI rotation
   - Grand rounds
   - Benign gynecology rotation
   - Oncology rotation
   - Other training (please specify)
4. What is your gender?
   - Male
   - Female
   - Rather not say

### FOR FEMALE RESPONDANTS ONLY

1. Do you think all female residents should consider oocyte freezing?
   - Yes
   - No
   - Unsure
2. Would you consider freezing your own oocytes to preserve your own fertility and postpone pregnancy?
   - Yes
   - No
   - Unsure
3. Would you consider freezing your own oocytes if you do not currently have a partner?
   - Yes
   - No
   - Unsure
4. If you would ever consider freezing your own oocytes, at what age would you consider freezing them?
   - 25 or under
   - 26-30
   - 31-34
   - 35-37
   - 38-40
   - 41-42
   - 43 or above
   - Unsure
5. If you see a patient who might benefit from oocyte-freezing, do you feel comfortable counseling her about this?
   - Yes
   - No
   - Unsure
6. What would you consider an affordable out-of-pocket cost for oocyte-freezing (with intent to freeze at least 10 oocytes)?
   - $2,000-$5,000
   - $5,000-$10,000
   - $10,000-$15,000
   - Unsure
7. Does your current employer offer an oocyte-freezing option as part of your benefits package?
   - Yes
   - No
   - Unsure
8. Would you prefer to be employed by an employer who offers oocyte freezing as part of benefits package?
   - Yes
   - No
   - Unsure

**FOR All RESPONDANTS**

1. What information about oocyte-freezing would you consider the most important to know as a prospective patient? (check all that apply)
   - Why do women freeze their oocytes?
   - What is the optimal age for oocyte freezing?
   - Are there health reasons to consider oocyte-freezing at a younger age?
   - How many years are frozen oocytes viable?
   - How much does an oocyte-freezing cycle cost?
   - What are the annual storage fees for frozen oocytes?
   - What is the average number of oocytes needed to obtain one pregnancy?
   - Can I donate unused frozen oocytes to another woman?
   - Can I donate unused frozen oocytes to research?
   - What happens if my frozen oocytes are lost due to error?
2. Would you consider freezing your oocytes if…? (check all that apply)
   - There is no effect on health of children born from frozen oocytes
   - You currently don’t have children
   - There is insurance coverage or employer financial support for the cycle
   - You are not currently have a partner
   - There is higher pregnancy rate using frozen oocytes than with natural or routine IVF treatment at advanced female age
   - Oocyte-freezing is available locally
3. Does your department have an IVF program?
   - Yes
   - No
   - Unsure
4. If yes, does your department offer oocyte-freezing to patients, on-site?
   - Yes
   - No
   - Unsure

1. How many weeks in your residency program are spent on an REI rotation?
   - None
   - 1 to 4 weeks
   - 5 to 8 weeks
   - 9 to 12 weeks
   - 13 to 16 weeks
   - 17 to 20 weeks
   - 21 or more weeks

1. What ethnicity do you primarily consider yourself?
   - African-American
   - Asian/Pacific Islander
   - American Indian/Alaska Native
   - Caucasian
   - Hispanic/Latino
   - Multiracial
   - Rather not say
2. What is your age?
   - 25 or under
   - 26-30
   - 31-34
   - 35-37
   - 38-40
   - 41-42
   - 43 or above

1. What degrees do you hold? (check all that apply)
   - MD
   - DO
   - PhD
   - MSc/MPH
2. What country is your residency program located?
   - United States
   - Canada
3. If in the United States, in what State is our program located?
   - Northeast Region (Connecticut, Maine, Massachusetts, New Hampshire, New York, Rhode Island, Vermont)
   - Mid-Atlantic Region (Delaware, Maryland, New Jersey, Pennsylvania, Virginia, District of Columbia, West Virginia)
   - Southeast Region (Alabama, Florida, Georgia, North Carolina, South Carolina, Tennessee)
   - Great Lakes Region (Illinois, Indiana, Iowa, Kansas, Kentucky, Michigan, Minnesota, Missouri, Nebraska, Ohio, Wisconsin)
   - Gulf Coast Region (Arkansas, Louisiana, Mississippi, Oklahoma, Texas)
   - Northwest Region (Colorado, Nevada, Utah, Washington)

1. How would you describe your program?
   - University hospital based
   - Community hospital based
   - Other

1. What is your current level of training in residency?
   - PGY-1
   - PGY-2
   - PGY-3
   - PGY-4
   - PGY-5 (Canada)
   - Fellow

1. What is your current relationship status?
   - Single
   - Married
   - Living with partner
   - Divorced
   - Widowed
   - Rather not say

1. Would you consider yourself?
   - Heterosexual
   - Homosexual
   - Bisexual
   - Rather not say

1. If you have a partner, would you be interested in freezing embryos than eggs?
   - Yes
   - No

1. Any additional comments regarding this survey?
